# Early cost-effectiveness analysis of screening for preeclampsia in nulliparous women: A modelling approach in European high-income settings

**DOI:** 10.1371/journal.pone.0267313

**Published:** 2022-04-21

**Authors:** Neily Zakiyah, Robin Tuytten, Philip N. Baker, Louise C. Kenny, Maarten J. Postma, Antoinette D. I. van Asselt, on behalf of IMPROvED Consortium

**Affiliations:** 1 Unit of PharmacoTherapy, Epidemiology & Economics (PTE2), Department of Pharmacy, University of Groningen, Groningen, The Netherlands; 2 Department of Pharmacology and Clinical Pharmacy, Faculty of Pharmacy, Universitas Padjadjaran, Bandung, Indonesia; 3 Center of Excellence in Higher Education for Pharmaceutical Care Innovation, Universitas Padjadjaran, Bandung, Indonesia; 4 Research & Development, Metabolomic Diagnostics, Little Island, Ireland; 5 College of Life Sciences, University of Leicester, Leicester, United Kingdom; 6 Department of Women’s and Children’s Health, the Faculty of Health and Life Sciences, University of Liverpool, Liverpool, United Kingdom; 7 Unit of Global Health, Department of Health Sciences, University Medical Center Groningen, University of Groningen, Groningen, The Netherlands; 8 Department of Economics, Econometrics & Finance, Faculty of Economics & Business, University of Groningen, Groningen, The Netherlands; 9 Unit of Patient Centered Health Technology Assessment, Department of Epidemiology, University Medical Center Groningen, University of Groningen, Groningen, The Netherlands; Holbaek Sygehus, DENMARK

## Abstract

**Background:**

Preeclampsia causes substantial maternal and perinatal morbidity and mortality and significant societal economic impact. Effective screening would facilitate timely and appropriate prevention and management of preeclampsia.

**Objectives:**

To develop an early cost-effectiveness analysis to assess both costs and health outcomes of a new screening test for preeclampsia from a healthcare payer perspective, in the United Kingdom (UK), Ireland, the Netherlands and Sweden.

**Methods:**

A decision tree over a 9-month time horizon was developed to explore the cost-effectiveness of the new screening test for preeclampsia compared to the current screening strategy. The new test strategy is being developed so that it can stratify healthy low risk nulliparous women early in pregnancy to either a high-risk group with a risk of 1 in 6 or more of developing preeclampsia, or a low-risk group with a risk of 1 in 100 or less. The model simulated 25 plausible scenarios in a hypothetical cohort of 100,000 pregnant women, in which the sensitivity and specificity of the new test were varied to set a benchmark for the minimum test performance that is needed for the test to become cost-effective. The input parameters and costs were mainly derived from published literature. The main outcome was incremental costs per preeclampsia case averted, expressed as an incremental cost-effectiveness ratio (ICER). Deterministic and probabilistic sensitivity analyses were conducted to assess uncertainty.

**Results:**

Base case results showed that the new test strategy would be more effective and less costly compared to the current situation in the UK. In the Netherlands, the majority of scenarios would be cost-effective from a threshold of €50,000 per preeclampsia case averted, while in Ireland and Sweden, the vast majority of scenarios would be considered cost-effective only when a threshold of €100,000 was used. In the best case analyses, ICERs were more favourable in all four participating countries. Aspirin effectiveness, prevalence of preeclampsia, accuracy of the new screening test and cost of regular antenatal care were identified as driving factors for the cost-effectiveness of screening for preeclampsia.

**Conclusion:**

The results indicate that the new screening test for preeclampsia has potential to be cost-effective. Further studies based on proven accuracy of the test will confirm whether the new screening test is a cost-effective additional option to the current situation.

## Introduction

Preeclampsia contributes significantly to the burden of maternal and perinatal morbidity and mortality worldwide [1, 2]. In high-income regions this burden is lower than in low and middle income countries, due to the availability of timely medical interventions that decrease the risks associated with pregnancy complicated by preeclampsia [3]. Nevertheless, preeclampsia and other hypertensive disorders remain responsible for approximately 13% of maternal deaths worldwide [2]. Early identification of preeclampsia is one of the important objectives of antenatal care in high-resource countries [4]. Effective screening, administered in the first half of pregnancy, would enable stratification of women according to their risk and thus inform the appropriate and tailored application of improved prevention, management and treatment of preeclampsia. This stratification would also reduce the cost of misclassification and lead to more efficient antenatal care in each group, resulting in potential cost-savings [5].

Screening for specific clinical risk factors in the first trimester of pregnancy, followed by low-dose aspirin prophylaxis for those at increased risk is recommended by several guidelines [6–8]. However, most of the recognised anticipated risk factors are associated with other comorbidities or with complications in previous pregnancy, and are thus not applicable to the majority of nulliparous pregnant women without overt risk factors [4, 9]. Consequently, the accuracy of clinical risk prediction for preeclampsia in nulliparous women is modest, and so (novel) biomarkers are sought for to assist in providing a personalized clinical risk profile to predict preeclampsia. Over the last decade, there has been considerable research into identifying potentially relevant biomarkers for preeclampsia; however, these novel biomarker-based screening tests have yet to be introduced in clinical practice [5, 10].

Previous economic evaluation studies show conflicting results as to the cost-effectiveness of preeclampsia screening. Several studies have suggested that additional biomarkers such as PP13, pregnancy-associated plasma protein-A (PaPP-A), placental growth factor (PIGF), along with uterine artery Doppler and biophysical feature combined with prophylactic aspirin for those classified as high risk of preterm preeclampsia (preeclampsia resulting in a iatrogenic delivery before 37 weeks of gestation) to be cost effective and even cost saving [11–13]. Other studies argue that screening may not be the most cost-effective option [14, 15]. Our previous systematic review on economic assessments of preeclampsia concluded that biomarker-based tests for preeclampsia screening have the potential to be a cost-effective approach for clinical practice, but their accuracy is a major driver for cost-effectiveness [10]. Routine screening for preeclampsia risk is potentially feasible, but only when accuracy is significantly improved [10].

An early cost-effectiveness study using decision modelling refers to analyses that are conducted early in the technology’s development process [16] and eventually could guide the predictive performance goals of a technology that is yet to be developed, or refine the specification of tests which are in the early stages of development [17–19]. We have previously proposed [20] that the ability to analyse multiple biomarkers simultaneously opens the possibility to either formulate a risk stratification test which is more effective in identifying a population at increased risk or a risk stratification test which is more effective in identifying a population at decreased risk. We tested this concept recently to identify and select biomarkers for a new proposed biomarker-based screening test [19].

The aim of this study was to develop an early cost-effectiveness model to assess both costs and health outcomes of a new screening test for preeclampsia compared to the current screening strategy from a healthcare payer perspective in four high-income European countries, i.e. United Kingdom (UK), Ireland, the Netherlands and Sweden.

In this analysis, we use decision-analytic modelling to identify the key drivers of cost-effectiveness and estimate at what value the new technology could still be cost-effective, in a number of exploratory simulated scenarios, and thus generate targets for the clinical performance specification for the novel test.

## Methods

### Model overview

**Fig 1:**
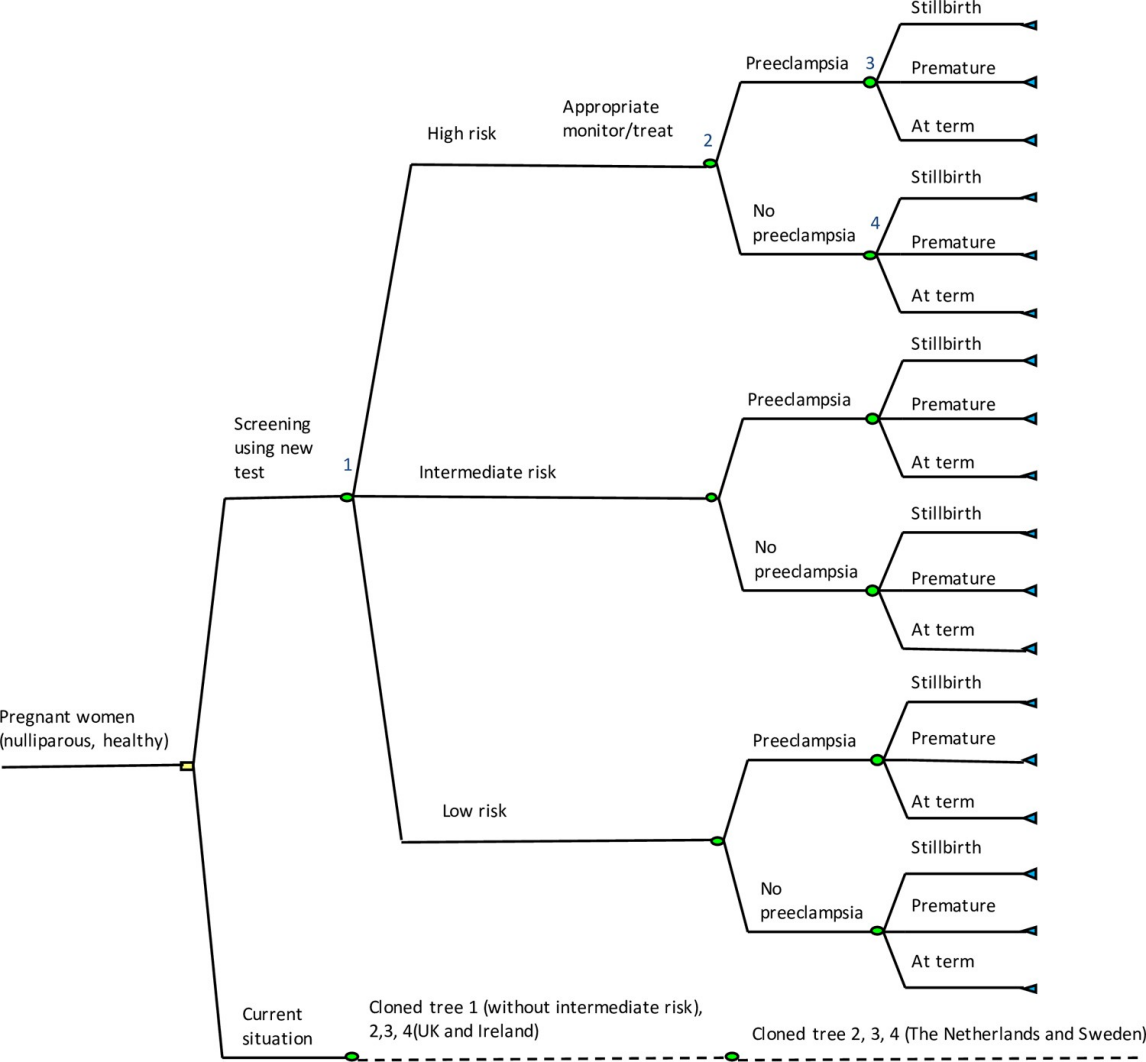
A decision tree comparing the new screening test strategy with the current screening test in UK, The Netherlands, Ireland, and Sweden. UK: United Kingdom.

The reporting standard for the evaluations followed the Consolidated Health Economic Evaluation Reporting Standards (CHEERS) statement [21]. A decision tree analytic simulation model, depicted in Fig 1, was constructed to investigate cost, potential health outcomes and cost-effectiveness of the new screening test for preeclampsia and the current screening strategy for healthy, first time mothers with a singleton pregnancy. The model was developed to follow a hypothetical cohort of 100,000 pregnant women through their pregnancy and recorded the health outcomes. The outcome of this model was expressed as incremental cost-effectiveness ratios (ICERs) per preeclampsia case averted for the new screening test as compared to the current situation. ICERs were estimated if the new screening test was more effective and more costly than the current situation. If the new screening test was more effective and less costly, it was defined as a “dominant” strategy. When the opposite occurred, it was categorized as a “dominated” strategy. The time horizon for the analyses was from the booking period until discharge of the mother and child from the hospital, therefore discounting of costs and outcomes was not necessary on account of the short time period for the analysis. The input parameters for the model were derived mostly from published literatures. Specific information i.e., frequency of potential increased visits for high risk group, average duration of hospitalization for preterm babies and prevalence of preeclampsia for each participating countries were obtained from a survey of expert opinion that was developed for healthcare professionals, as a part of IMproved Pregnancy Outcomes by Early Detection (IMPROvED) project [17–19, 22] and provided in Table 1. Details on the survey are provided in S1 Table.

**Table 1:** Input parameters.

Input data	Value	Reference
**Probability of preeclampsia**		
High-risk group via current screening (UK)	1 in 20	[17, 38]
Low-risk group via current screening (UK)	1 in 40	[17, 38]
High-risk group via current screening (Ireland)	1 in 16	[17, 38]
Low-risk group via current screening (Ireland)	1 in 31	[17, 38]
High-risk group via new test strategy	1 in 6	[29]
Low-risk group via new test strategy	1 in 100	[20, 28, 29]
**Effectiveness of monitor/treat for high-risk group**		
RR with aspirin for high-risk women (95% CI) (base-case)	0.88 (0.49–0.97)	[31, 36, 37]
RR with aspirin for high-risk women (95% CI) (best-case)	0.57 (0.43–0.75)	[37]
**Frequency of increased visits (for high-risk group)**		
Obstetrician	4 more visits	Survey
Ultrasounds	2 more visits	Survey
Duration of preventive treatment	25 weeks	Assumption
**Delivery**		
Home birth proportion for low-risk women (The Netherlands)	7.5%	[40]
Proportion of normal delivery in pregnancy without preeclampsia	87%	Estimation*
Proportion of caesarean section (c-section) delivery in pregnancy without preeclampsia	13%	[41]
Proportion of normal delivery in preeclampsia	59%	Estimation*
Proportion of c-section delivery in preeclampsia	41%	[41]
**Birth outcomes in pregnancy without preeclampsia**		
Proportion of term birth	95.27%	Estimation**
Proportion of premature birth	4.47%	[42]
Proportion of stillbirth	0.27%	[43]
**Birth outcomes in pregnancy with preeclampsia**		
Proportion of term birth	71.84%	Estimation**
Proportion of premature birth	22.49%	[42]
Proportion of stillbirth	5.67%	[36]
**Delivery outcome (for live births)**		
Duration of hospitalization for preterm babies	18 days (6 days in NICU and 12 days in neonatal ward)	Survey and [40]

RR: Relative risk; CI: Confidence Interval

*In the model, delivery was assumed to be only categorized as normal and c-section, therefore the proportion of normal delivery was assumed to be the remaining proportion of c-section delivery.

**it was assumed that the birth outcomes comprised only term birth, premature birth and stillbirth, therefore the estimation of term birth was derived as a remaining proportion of premature and stillbirth.

#### Ethical considerations

Some input parameters for the model were derived from a survey from the IMPROvED project (approved by The Clinical Research Ethics Committee of Cork Teaching Hospitals, approval number ECM5 (3) 06/08/13) and written informed consent was obtained from all healthcare professionals who participated in the survey. Collection of data from the survey complied with standardised procedures in all participating centres. For the model outcomes, since the model used a hypothetical cohort, there was no involvement of patients and public in the study and no patient interviews were conducted for the model outcomes.

#### Definition preeclampsia

Preeclampsia was defined as the new onset of high blood pressure (persistent blood pressure ≥140 mmHg systolic and / or diastolic ≥90 mmHg) that occurred after 20 weeks of gestation with the presence of multisystemic dysfunction with or without proteinuria, in a previously normotensive woman [7, 23–26].

#### Current situation

In the UK and Ireland, pregnant women with more than one moderate risk factor for developing preeclampsia are recommended to take low dose aspirin prophylaxis (75–150 mg per day) from 12 weeks until birth. The moderate risk factors are: i.e. first pregnancy, age 40 years or older, pregnancy interval of more than 10 years, multi-fetal pregnancy, body mass index (BMI) of 35 kg/m^2^ or more at first antenatal visit and family history of preeclampsia [7, 25].

In contrast, The Netherlands and Sweden do not explicitly formulate a recommendation applicable to nulliparous women, and only emphasise screening and treatment recommendations for women at increased risk i.e. pregnant women with co-morbidities such as chronic hypertension and kidney disease, diabetes mellitus, and autoimmune disease [23, 24]. Additionally in Sweden, those with three or more aforementioned moderate risk factors, should also be considered to take low dose aspirin [27]. Thus, for the UK and Ireland we determined the maternal risk factor screening and subsequent treatment to be the current screening strategy, and we assumed similar situation with no screening for preeclampsia applicable for nulliparous in The Netherlands and Sweden.

In order to collect information about regular antenatal care in the different participating countries, an online survey on the management of healthy pregnancies, pregnancies at increased risk of preeclampsia and preeclampsia pregnancies was developed for healthcare professionals [17]. This survey identified, beside treatment recommendations, that increased monitoring in the form of more frequent contacts with healthcare professionals was also necessary for the management of those identified as at high-risk for developing preeclampsia. The survey details are provided in S1 Table.

#### New screening test strategy

In the context of the model, we defined the new screening test as a novel predictive blood test using metabolomic biomarkers that is being developed as part of the IMPROvED project. In addition, the new test strategy would stratify nulliparous women into risk categories based on the risks observed in second (and further) pregnancies. More specifically, nulliparous women classified as high-risk according to the new test strategy would have a risk of at least 1 in 6, which is the risk of recurrence in a multiparous woman after preeclampsia in a preceding pregnancy. It is noted that this corresponds to setting the minimum Positive Predictive Value (PPV) for the test to 1/6 or 0.166 in accordance with Thomas et al. [20]. Women classified as low-risk according to the new test strategy would have a risk of at most 1 in 100, which is the risk of preeclampsia in woman’s second pregnancy when her first pregnancy was without complications [20, 28, 29]. This is equivalent to setting the minimum Negative Predictive Value (NPV) to 0.99. Since not all tested women will be either ruled in to be at high-risk, or ruled out and be classified as low-risk, the remainder would be classified as intermediate-risk. The estimates for the number of preeclampsia cases in those not identified as high-risk or low-risk were based on sensitivity and specificity of the test. The risk stratification was assumed to be accompanied by several follow-up strategies for each group. We assumed that women at high-risk would receive the same treatment as pregnant women with risk factors, including increased monitoring and treatment as recommended i.e. low dose aspirin prophylaxis. Women classified as intermediate-risk would receive similar antenatal care as in the current screening strategy, and those classified as low-risk would receive the care model pertinent to second pregnancies, i.e. a reduction in number of antenatal appointments by 30% [30]. In addition, the provision of low dose aspirin prophylaxis was only applied in the high-risk group, but not in intermediate and low-risk groups.

### Model structure

In the model, routine antenatal care was implemented early in pregnancy or in the booking period (which occurred around 8–12 weeks of gestation). This was considered to be the time for the doctor or midwife to confirm the pregnancy and to conduct a basic assessment of the pregnant women. The decision node (i.e. the square node in Fig 1) represents the comparison between the following two strategies:

1. Screening all pregnant women in all participating countries using the new screening test in combination with maternal risk factors at 15 weeks of gestation to determine their risk of developing preeclampsia. The testing time point was chosen based on previous reported findings [4]. The high-risk group was directed to be in the increased monitoring group, with more frequent visits to obstetrician and/ or midwives and prophylactic treatment with low dose aspirin prophylaxis. Evidence from numerous randomized controlled trials and meta-analysis has confirmed that daily low-dose aspirin could reduce the overall risk for preeclampsia in women at increased risk of developing preeclampsia [31–35]. Effectiveness of low dose aspirin prophylaxis was incorporated in the model with associated relative risk estimates derived from published studies [31, 36, 37]. The estimates regarding the increases in visit frequency, and the differences in the choice of healthcare professionals who will perform the further (post-test) pregnancy monitoring (obstetrician, general practitioner, midwife, etc), were based on the results from the aforementioned survey. It was estimated that women classified as being at high-risk should have four extra visits from obstetricians and two extra ultrasound scan appointments. In the absence of an effectiveness measure for the increased monitoring, we assumed that the effectiveness of prophylaxis treatment comprised the effect of the increased visits as well, so no additional effects were calculated for the increased monitoring *per se*.
2. The current strategy i.e. regular antenatal care in UK, Ireland, the Netherlands and Sweden. As mentioned previously, screening using maternal risk factors and treatment with low-dose aspirin was assumed to be the current screening strategy in UK and Ireland. The assumption was that pregnant women were screened in the booking period and stratified to be either in the high-risk or to be in the low-risk group. The high-risk group received the same management as those in the new test strategy, while the low-risk group received regular antenatal care. The country-specific prevalence of preeclampsia was used to estimate the probability of developing preeclampsia in the low and high-risk group with current maternal risk factor based screening in UK and Ireland. Moreover, we assumed that for the Netherlands and Sweden, in alignment with the guidelines in these countries, pregnant women received regular antenatal care from the booking period and would be detected as having preeclampsia if signs occurred after 20 weeks of gestation.

The prevalence of preeclampsia in the four participating countries was derived from IMPROvED data and were estimated to be 2.9% in UK, 3.2% in the Netherlands, 3.7% in Ireland and 1.7% in Sweden. The model also estimated pregnancy outcomes, i.e. term birth, premature birth, and stillbirth, for pregnancies with and without preeclampsia. Preeclampsia is associated with higher rates of caesarean deliveries and preterm birth, which consequently were also more likely to require hospitalizations for both mother and their offspring, as well as a higher utilization of the neonatal intensive care unit (NICU) [12, 38]. For UK, Ireland, and Sweden, we assumed that all deliveries occurred in hospital. For the Netherlands the situation is different, as home birth is part of the established Dutch maternity care system for low-risk pregnant women without complications [39]. Hence for the Netherlands, we took into account the proportion of home-births for nulliparous, low-risk pregnant women without preeclampsia. Furthermore, the mode of delivery, either vaginal delivery or caesarean section, as well as the probabilities for the different potential pregnancy outcomes for both preeclampsia and non-preeclampsia pregnancies in all countries were included in the estimation. Table 1 summarizes the input parameters for the model.

### Costs estimation

The healthcare provider perspective was used for the analysis, therefore we included only direct medical costs. The country-specific costs were estimated for costs of regular antenatal care, cost of increased monitoring and preventive treatment for the high-risk group, costs of preeclampsia cases including hospitalization and treatment, costs of delivery, and costs of neonatal intensive care unit for preterm birth.

The cost of regular antenatal care consisted of a set of appointments for low-risk nulliparous women, including appointment visits by midwife/ clinician, ultrasounds, screening and fetal assessment. In the screening using new test strategy, the price for the test was set to a potential cost of € 150. We assumed that, as the current screening strategy takes places within regular antenatal care, its costs are already accounted for in the UK and Ireland. The cost of increased monitoring and preventive treatment for the high-risk group comprised costs of more frequent visits to obstetrician and/or midwives, and costs for daily low dose aspirin and calcium supplementation. Pregnant women at increased risk of developing preeclampsia are recommended to take daily low dose aspirin from the time of the assessment until birth [7, 25]. We assumed comparable timing for both the new test strategy and the current screening i.e. at 15 weeks of gestation; therefore the duration for both increased monitoring and preventive treatment was estimated to be 25 weeks. As the recommended dose of aspirin for preventive treatment was 75mg daily [34], the unit price of a 75mg aspirin tablet was used for cost estimation [44, 45].

Due to a lack of country-specific data on cost of preeclampsia care, we assumed that the cost would be the same in all four participating countries. The estimation was based on a recently reported estimation of preeclampsia care in Ireland [46], including hospital admissions costs (hospital admissions ante- and postpartum weighted by the average length of stay for the mothers) as well as treatment for preeclampsia care, and excluding cost of delivery. Moreover, we also included country-specific costs of either vaginal delivery or caesarean section and cost of hospitalization and NICU admission in case of preterm birth, for all pregnancies with or without preeclampsia. The cost for neonatal hospitalization was estimated by weighing the cost with the average number of days preterm babies would spend in both NICU and neonatal ward. From the survey, we derived the average of 14–20 hospitalization days for premature babies born between 34–37 weeks. From this estimation, 18 days of neonatal hospitalization that comprised 6 days in NICU and 12 days in neonatal ward was used for the analysis [40].

All costs were adjusted to Euro 2020 using inflation rates and official exchange rates from the World Bank annual consumer index. Details on included costs are available on Table 2.

**Table 2:** Estimated costs (Euro, 2020).

Costs	Countries				Reference			
	UK	The Netherlands	Ireland	Sweden	UK	The Netherlands	Ireland	Sweden
Cost of regular antenatal care	€ 1,263	€ 662	€ 355	€ 557	[47]	[48]	[46]	[49]
Cost of new screening test	€ 150	€ 150	€ 150	€ 150				
**Costs of monitor/treat for high-risk group**								
Obstetrician	€ 154	€ 85	€ 143	€ 75	[47]	[50]	[51]	[52]
Midwives (per hour)	€ 93	€ 42	€ 33	€ 37	[47]	[48, 53]	[51]	[52]
Ultrasounds (per visit)	€ 126	€ 47	€ 11	€ 101	[47]	[48]	[51]	[49]
Aspirin (25 weeks)	€ 2	€ 2	€ 2	€ 2	[44]	[44]	[44]	[44]
Calcium supplement (25 weeks)	€ 19	€ 19	€ 19	€ 19	[45]	[45]	[45]	[45]
**Delivery outcome costs (for mothers)**								
Cost of preeclampsia care (including hospitalization, treatment)	€ 2,967	€ 2,967	€ 2,967	€ 2,967	[46]	[46]	[46]	[46]
Normal delivery	€ 2,783	€ 2,373	€ 704	€ 2,319	[47]	[54]	[46]	[49]
C-section delivery	€ 5,104	€ 4,531	€ 1,058	€ 4,755	[47]	[54]	[46]	[49]
Home birth	NA	€ 557	NA	NA	NA	[48]	NA	NA
**Delivery outcome costs (for live births)**								
Cost of NICU per day	€ 1,538	€ 1,282	€ 910	€ 2,697	[47]	[54]	[55]	[49]
Cost of neonatal ward (normal care) per day	€ 566	€ 360	€ 255	€ 610	[47]	Estimation and [54]	Estimation and [55]	[49]

### Analyses

Exploratory scenario analyses were performed where we independently varied the sensitivity and specificity of the new test (at PPV 1 per 6 and NPV 99 per 100) in 25 plausible scenarios, ranging from 35% until 75%. The analyses were used to set a benchmark for the minimum new test performance that is needed for it to become cost-effective compared to current screening.

In the exploratory analyses, we performed base-case analyses where we used a rather modest effectiveness of aspirin prophylaxis [31, 36, 37]. Higher effectiveness of prophylactic aspirin, based on a more recent study, was used in best- case analyses [37].

Probabilistic sensitivity analyses (PSA) were performed in which incremental costs and outcome of preeclampsia cases were estimated in a Monte Carlo simulation with 10,000 iterations. We pre-selected five appropriate scenarios to represent lowest, highest and modest combination of sensitivity and specificity (within a 35%-75% range) to be assessed in the PSA i.e. scenario 1 (35% sensitivity, 75% specificity), scenario 3 (55% sensitivity, 75% specificity), scenario 13 (55% sensitivity, 55% specificity), scenario 21 (35% sensitivity, 35% specificity) and scenario 25 (75% sensitivity, 35% specificity). All other relevant parameters were varied simultaneously according to the reported 95% confidence interval and from the appropriate distributions of the input parameters. Parameters involved in PSA are effectiveness of aspirin, proportion of normal delivery and caesarean section in pregnancy with and without preeclampsia, proportion of birth outcomes in pregnancy with and without preeclampsia (i.e. term birth, premature birth and stillbirth) and costs. Log normal distribution was used for aspirin effectiveness, beta distributions were used for proportion of delivery as well as proportion of birth outcomes in pregnancy, and gamma distribution was fitted for costs.

Cost-effectiveness planes and acceptability curves were generated from the Monte Carlo simulation to present the probability of the new test strategy to be cost-effective over a range of willingness-to-pay thresholds i.e. €10,000, €30,000, €50,000 and €100,000 per preeclampsia case averted, that were used previously in a threshold analysis to determine the cost-effectiveness of a preeclampsia test [14].

### Results

Table 3 depicts the results of the base case (using moderate effectiveness of aspirin prophylaxis) and best case (using the more optimistic effectiveness) analyses in exploratory scenarios.

**Table 3:** Cost-effectiveness of new test strategy versus current screening strategy in exploratory scenario analyses in four participating countries, i.e. UK, The Netherlands, Ireland and Sweden.

Scenario	Sensitivity*	Specificity*	ICER per preeclampsia cases averted							
			UK		The Netherlands		Ireland		Sweden	
			Base-case	Best-case	Base-case	Best-case	Base-case	Best-case	Base-case	Best-case
**Scenario 1**	35%	75%	Dominant	Dominant	€ 19,153	€ 1,111	€ 67,364	€ 897	€ 53,104	€ 9,134
**Scenario 2**	45%	75%	Dominant	Dominant	€ 18,445	€ 914	€ 43,243	€ 1,652	€ 45,172	€ 6,920
**Scenario 3**	55%	75%	Dominant	Dominant	€ 17,994	€ 788	€ 36,677	€ 2,088	€ 40,124	€ 5,512
**Scenario 4**	65%	75%	Dominant	Dominant	€ 17,682	€ 701	€ 33,615	€ 2,372	NA	NA
**Scenario 5**	75%	75%	NA	NA	€ 17,453	€ 637	€ 31,843	€ 2,571	NA	NA
**Scenario 6**	35%	65%	Dominant	Dominant	€ 33,609	€ 5,145	€ 98,558	€ 3,280	€ 76,356	€ 15,623
**Scenario 7**	45%	65%	Dominant	Dominant	€ 29,688	€ 4,051	€ 56,592	€ 3,397	€ 63,257	€ 11,967
**Scenario 8**	55%	65%	Dominant	Dominant	€ 27,193	€ 3,355	€ 45,168	€ 3,464	€ 54,921	€ 9,641
**Scenario 9**	65%	65%	Dominant	Dominant	€ 25,466	€ 2,873	€ 39,841	€ 3,508	NA	NA
**Scenario 10**	75%	65%	Dominant	Dominant	€ 24,200	€ 2,520	€ 36,757	€ 3,538	NA	NA
**Scenario 11**	35%	55%	Dominant	Dominant	€ 48,065	€ 9,180	€ 129,753	€ 5,664	€ 99,608	€ 22,112
**Scenario 12**	45%	55%	Dominant	Dominant	€ 40,932	€ 7,189	€ 69,941	€ 5,142	€ 81,342	€ 17,014
**Scenario 13**	55%	55%	Dominant	Dominant	€ 36,393	€ 5,922	€ 53,660	€ 4,840	€ 69,718	€ 13,770
**Scenario 14**	65%	55%	Dominant	Dominant	€ 33,250	€ 5,045	€ 46,066	€ 4,644	€ 61,670	€ 11,525
**Scenario 15**	75%	55%	Dominant	Dominant	€ 30,946	€ 4,402	€ 41,672	€ 4,506	NA	NA
**Scenario 16**	35%	45%	Dominant	Dominant	€ 62,521	€ 13,214	€ 160,948	€ 8,047	€ 122,860	€ 28,601
**Scenario 17**	45%	45%	Dominant	Dominant	€ 52,176	€ 10,327	€ 83,290	€ 6,886	€ 99,427	€ 22,061
**Scenario 18**	55%	45%	Dominant	Dominant	€ 45,592	€ 8,490	€ 62,151	€ 6,216	€ 84,514	€ 17,900
**Scenario 19**	65%	45%	Dominant	Dominant	€ 41,034	€ 7,218	€ 52,292	€ 5,780	€ 74,191	€ 15,019
**Scenario 20**	75%	45%	Dominant	Dominant	€ 37,692	€ 6,285	€ 46,586	€ 5,473	NA	NA
**Scenario 21**	35%	35%	Dominant	Dominant	€ 76,977	€ 17,248	€ 192,143	€ 10,431	€ 146,112	€ 35,090
**Scenario 22**	45%	35%	Dominant	Dominant	€ 63,419	€ 13,465	€ 96,639	€ 8,631	€ 117,511	€ 27,108
**Scenario 23**	55%	35%	Dominant	Dominant	€ 54,792	€ 11,057	€ 70,642	€ 7,592	€ 99,311	€ 22,029
**Scenario 24**	65%	35%	Dominant	Dominant	€ 48,818	€ 9,390	€ 58,518	€ 6,916	€ 86,711	€ 18,513
**Scenario 25**	75%	35%	Dominant	Dominant	€ 44,438	€ 8,168	€ 51,501	€ 6,440	€ 77,471	€ 15,934

*Sensitivity and specificity of the new screening test

ICER: Incremental cost-effectiveness ratio, UK: United Kingdom, NA: not applicable

Dominant: new test strategy is more effective (better health outcomes) with lower cost compared to current screening.

NA indicates that the combination of sensitivity and specificity is not applicable due to low prevalence

UK base-case results showed that the new test strategy would be cost-saving and thus be a dominant option as opposed to the current screening in all scenarios, with less total costs and more preeclampsia cases averted.

For the Netherlands, when using a willingness to pay threshold of €10,000 per preeclampsia case averted, the new test strategy would not be considered cost-effective. When using a threshold of €30,000, 36% of scenarios (9 out of 25 scenarios) were cost-effective, with minimum combinations of sensitivity and specificity of either 35% and 75% or 65% and 65%, respectively. If a threshold of €50,000 was used, the majority of scenarios (80%) would be cost-effective, with minimum combinations of sensitivity and specificity of 35% and 65%, 45% and 55% or 55% and 45%, respectively. All scenarios would be cost-effective at willingness to pay thresholds of €100,00 or more per preeclampsia case averted.

In Ireland, less than half (40%) out of 25 scenarios had ICERs below €50,000 per preeclampsia case averted, suggesting that the new test strategy was most likely not cost-effective compared to the current screening strategy if a threshold below €50,000 was used. The minimum combinations of sensitivity and specificity for the new test strategy to be cost-effective under willingness to pay below €50,000 were either 45% and 75% or 55% and 65% for both combinations of sensitivity and specificity. At the €100,000 threshold, 88% of scenarios would be considered cost-effective, with sensitivity above 35%. In Sweden, a similar trend was observed, as the vast majority of scenarios were only considered cost-effective at the €100,000 threshold.

In best case analyses, where higher effectiveness of prophylactic aspirin was used to inform the model, the overall ICER in all four participating countries appeared to improve, as expected. In UK, similar to base-case results, all scenarios resulted in dominance of the new test strategy over current screening, i.e. the new test strategy most likely would save costs and prevent preeclampsia cases. In the Netherlands, the new test strategy would appear to be mostly cost effective using the lowest willingness to pay threshold of €10,000. A comparable trend was also observed in Ireland. In Sweden, all scenarios were cost-effective at a threshold of €30,000 or higher per preeclampsia case averted.

### Probabilistic sensitivity analysis

**Fig 2:**
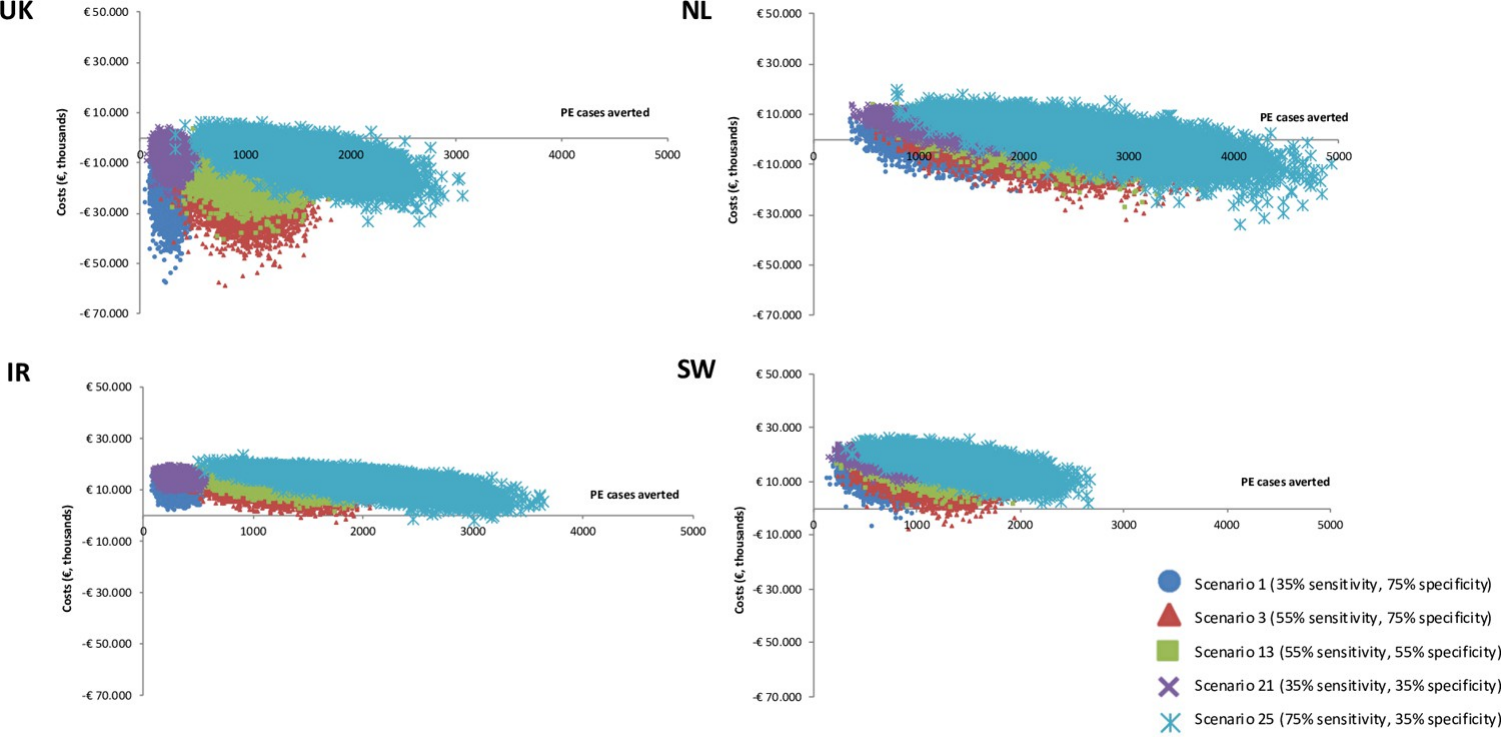
Cost-effectiveness planes of the new screening test for preeclampsia versus current screening strategy in base-case scenario analyses in four participating countries. UK: United Kingdom, NL: The Netherlands, IR: Ireland, SW: Sweden, PE: preeclampsia.

Fig 2 shows the cost-effectiveness planes of five selected scenarios i.e. scenario 1, scenario 3, scenario 13, scenario 21, and scenario 25 in base case. The results demonstrated that all estimates in all participating countries were scattered within the northeast or southeast quadrants, meaning that the new test strategy was certainly more effective, although in terms of costs, the probability distribution ranged from the test scenario being less expensive than current practice to being costlier. Overall, the trend in the PSA results suggested that higher sensitivity indicated more preeclampsia cases averted but also higher cost. Whereas higher specificity led to fewer preeclampsia cases averted but more savings.

**Fig 3:**
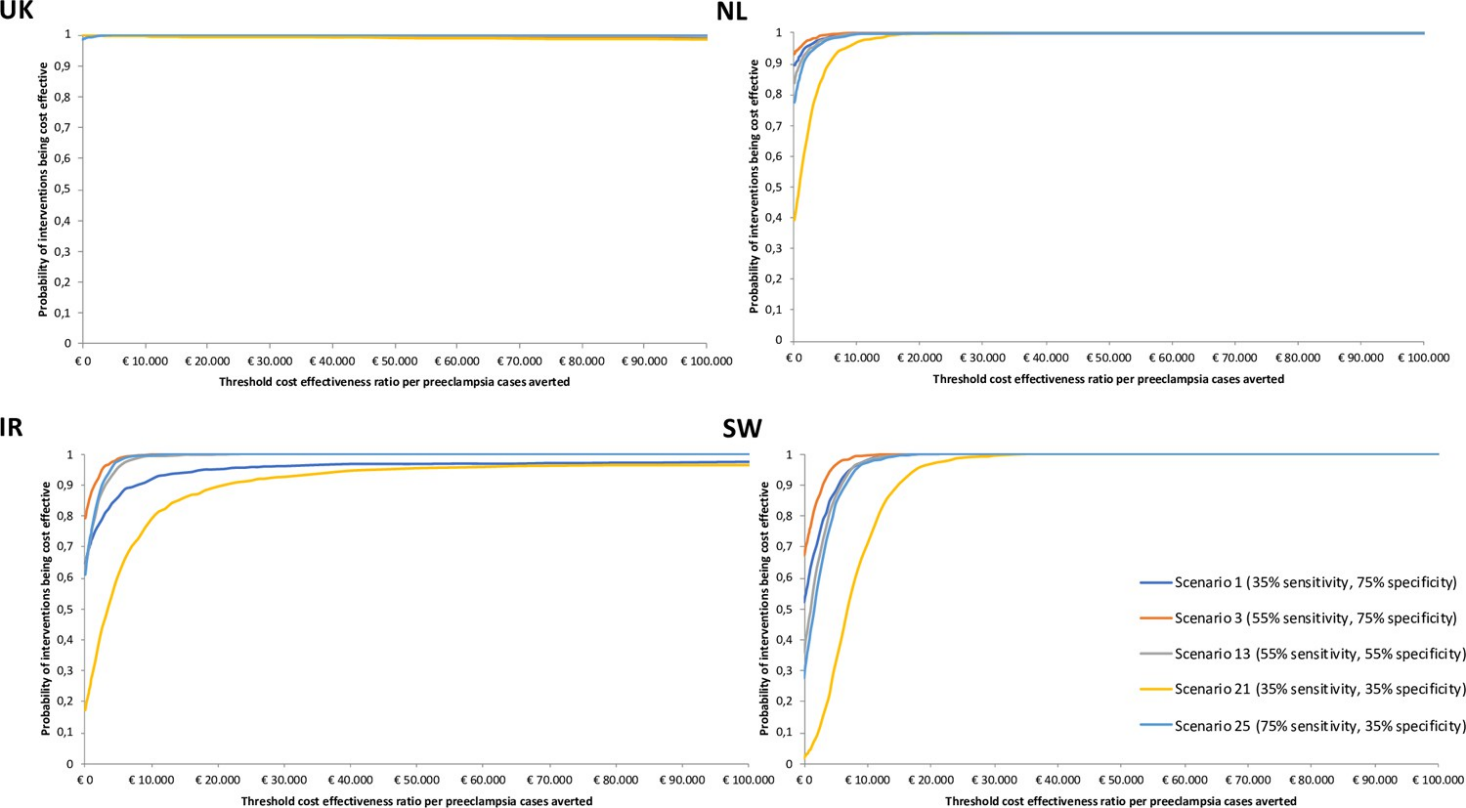
Cost-effectiveness acceptability curves for the new screening test in best-case scenario analyses in a different willingness to pay thresholds ranging from €10,000 - €100,000 per preeclampsia cases averted, in four participating countries. UK: United Kingdom, NL: The Netherlands, IR: Ireland, SW: Sweden.

Similar to deterministic results, the best-case PSA indicated improved overall ICERs in all countries, with more averted preeclampsia cases. Fig 3 depicts the cost-effectiveness acceptability curves (CEAC) for the new test strategy in a different range of willingness to pay thresholds from €10,000 - €100,000 in best-scenario.

## Discussion

Our exploratory scenario results indicate that there are differences in cost-effectiveness in four participating countries. Overall, there are several significant driving factors for cost-effectiveness of screening for preeclampsia i.e. aspirin effectiveness, prevalence of preeclampsia, accuracy of the new screening test and cost of regular antenatal care.

The analyses using the more optimistic effectiveness of prophylactic aspirin resulted in an improved overall ICER in the four participating countries compared to the ICERs obtained in the analyses using moderate effectiveness. The data to estimate moderate effectiveness of aspirin with relative risk of 0.88 is generated from several studies and meta-analyses which pooled results of studies applying various doses ranging from 60–150 mg daily [31, 32, 36, 37, 56], and the more optimistic effectiveness with relative risk of 0.57 was derived from a recent meta-analysis assessing the role of prophylactic aspirin for the prevention of preeclampsia [37]. The results of this study suggested that prophylactic treatment initiated before 16 weeks of gestation has a dose-response effect [37]. The previous study suggested limited benefit of lower dose aspirin (i.e. 60 mg daily) initiated in the first trimester of pregnancy for preeclampsia prevention [57]. However, there is growing evidence that the use of aspirin >75 mg/day started before 16 weeks of gestation for women identified as high risk can effectively reduce the prevalence of preeclampsia [37, 58, 59], and more particularly preterm and severe preeclampsia [37]. Although the aetiology of preeclampsia remains unclear, impaired placentation in the first 16 weeks of pregnancy is associated with an increased risk of the subsequent development of preeclampsia (particularly early onset disease) and the related condition of intrauterine fetal growth restriction (IUGR) [60, 61]. Numerous randomized controlled trials [35, 62] and subsequent meta-analyses [33, 37, 57] have reported that the risk of early onset preeclampsia and IUGR is reduced by low dose aspirin prophylaxis initiated before 16 weeks. Conversely, term preeclampsia is likely due to a different pathologic cause and less likely to be ameliorated by low dose aspirin than preterm preeclampsia [37, 63]. The updated National Institute for Clinical Excellence (NICE) and other international guidelines for hypertension in pregnancy advise women at high risk of preeclampsia to take 75–150 mg of aspirin daily from the first trimester of pregnancy until the delivery of the baby [7, 64].

The prevalence of preeclampsia also had a sizeable impact on cost-effectiveness results, in the sense that the lower the prevalence (e.g. Sweden), the less cost-effective universal screening would be. In this study, we used the prevalence of preeclampsia based on IMPROvED data. The real-world prevalence might be higher than prevalence observed in IMPROvED, as the trial population may not be fully representative of the general population with respect to risk factors for preeclampsia. Another driving factor for the cost-effectiveness is, obviously, the accuracy of the new screening test. The preeclampsia detection rate in nulliparous of current screening using maternal characteristics is estimated to be 24.8% at about 11.5% False Positive Rate (FPR) or 88.5% Specificity [38]. For the same FPR of 11.5%, maternal clinical factors combined into a multivariable regression model resulted in a slightly higher detection rate of 31% for preeclampsia at any gestation in nulliparous, and 35.9% and 41.6% for preeclampsia at <37 weeks of gestation, and <34 weeks of gestation, respectively [38]. The combination of maternal clinical factors, including mean arterial pressure (MAP) and uterine artery pulsatility index (UtA-PI), and also maternal biomarkers such as pregnancy-associated plasma protein and PIGF was estimated to be 54.2% for preeclampsia at any gestation for 9.2% FPR with 76.1% and 42.4% detection rate for preterm and term preeclampsia, respectively [65], yet it can be inferred from the same data that FPR will be higher when considering nulliparous only. When the accuracy of the new test increases, the number of low-risk women who may receive a reduced number of antenatal appointments would increase, and those who may receive unnecessary increased monitoring would decrease, which essentially escalates the likelihood of the new screening test becoming cost-effective.

The cost of regular antenatal care is also an important driving factor for cost-effectiveness. The higher these costs are, the higher the probability that screening is cost-effective because expensive regular care would leave more room for cost saving in those at low risk. This was reflected in the UK analysis, where the cost of regular antenatal care was the highest of the four participating countries. For the UK, dominance of the new test strategy was observed in all scenarios even in the base case scenarios where modest effectiveness of prophylactic aspirin was used. In contrast, for Ireland, which was the country with the lowest cost of regular antenatal care, ICERs were unfavourable at a willingness to pay threshold below €50,000, which was all the more striking since the prevalence estimate of preeclampsia for Ireland was higher than for the UK. The set of appointments in regular antenatal care between countries was also different. The number of appointment visits by midwife or clinician and ultrasound was also diverse between countries. For instance, 10 antenatal appointments were provided in the UK for low-risk nulliparous women [47], while in Ireland, regular antenatal care comprises on average of 6 visits [46]. However, the estimation of regular antenatal cost for Ireland was solely based on best-available data from the perspective of public healthcare [46]. Ireland currently has dual insurance system, in which women may choose either public or private maternity care. It was estimated that approximately one-third of pregnant women in Ireland receive private care [66].

Based on our previous systematic review [10], there were only very few published cost-effectiveness analysis studies on screening and diagnosis of preeclampsia [12, 14, 67], especially on screening [12, 14]. The results from previous studies vary reflecting different screening interventions. A study by Meads et al. [14] showed that screening was not cost-effective. However, the interventions assessed in this study left out potential novel biomarkers with improved accuracy [14]. Another study [12] indicates that screening for preeclampsia with additional biomarkers i.e., PP13 has a potential to be cost-effective, although uncertainties remain on some particular important parameters, such as the prevalence of preeclampsia, the effectiveness of prevention strategies and screening accuracy. This study also assumed less impact of low-dose aspirin intervention in reducing the prevalence of preeclampsia. The more recent cost-effectiveness of first trimester screening in Canada and Australia suggested that the implementation of early screening program coupled with early intervention with aspirin prophylaxis has the potential to avert significant number of preeclampsia cases, thus resulting in cost-saving [11, 13]. The Canadian and Australian studies included a multivariate model combining MAP, and biomarkers PaPP-A and/ or PIGF, and uterine artery Doppler, with early initiation of low dose aspirin for those categorized as high risk [11, 13]. Another recent cost-effectiveness study suggested that universal aspirin use regardless of women’s specific risk was dominant compared to other strategies, i.e., no aspirin prophylaxis, aspirin prophylaxis given to women based on risk stratification obtained from biomarker and ultrasound measures, and aspirin prophylaxis given to women’s risk status based on maternal history risk factors [15]. However, this study did not consider the consequences of the potential reduction in preterm delivery, which undeniably contributes to higher healthcare costs in the case of preeclampsia [68, 69]. Another limitation was that the strategy to give universal aspirin to all pregnant women regardless of their risk, exposed these vulnerable groups to unnecessary drug exposure. The potential complication that might occur includes postpartum haemorrhage and increased risk of perinatal complications [62].

The current early analysis can contribute to estimating the cost-effectiveness of a new test strategy in development and provide valuable insights on the potential parameters that drive the cost-effectiveness, before the implementation of the new technology in clinical practice. This can be important in refining test characteristics during further product development as well as future research when more detailed parameters are readily available [70, 71]. For instance, we note that this CEA indicates that from a healthcare resources optimisation perspective there may be scope for tests that solely focus on identifying women at decreased risk. This uncommon viewpoint certainly warrants further exploration in terms of product development potential.

In addition, the multi-country design with disparities in terms of prevalence of preeclampsia, costs of antenatal care and in terms of the current screening situation, allow us to generate a more comprehensive analysis on both costs and health consequences of the intervention in diverse settings. We also are able to highlight the driving factors of the analysis that are applicable in the various settings.

Inevitably, this study has several limitations. In common with other early cost-effectiveness analysis studies, data for several input parameters were incomplete. In our study, data regarding current care and the new test was lacking, therefore we synthesized some of the input parameter and costs data for current antenatal care from multiple data sources and also made assumptions, supported by expert opinion, regarding the probability of developing preeclampsia in the new screening test strategy. In addition, based on the data available to us, pricing of antenatal care was found to be very heterogeneous, resulting in substantial cost differences for antenatal care between countries, even when resource use was more or less comparable. We also assumed that the cost of preeclampsia care would be similar in all participating countries due to lack of country-specific data on this. This assumption can be a potential limitation, as this cost can be varied among countries. Although we incorporated the uncertainty of all cost estimates in the PSA, this has probably only partly addressed the structural issue of different pricing approaches between countries. Furthermore, we were not able to differentiate the effect of prophylactic aspirin between early and term preeclampsia in the model, which may lead to bias, although we tried to address this by using two different estimates (moderate and optimistic) for our analyses.

Additionally, we also did not consider long-term consequences of preeclampsia and the broader perspective, taking into account indirect costs relevant to the society such as productivity loss, in the model. There is growing evidence that preeclampsia is associated with later health consequences, especially for the mothers [72, 73]. The inclusion of these consequences and also a more comprehensive perspective might result in more favourable cost-effectiveness. Another potential limitation of our study might be the issue of implementation of the downgraded care pathway for low-risk pregnancies. In the model, we stratified nulliparous women into risk groups comparable to the risks as found in women in second or further pregnancies. Consequently, we assumed that those identified as low-risk would receive a 30% reduction in the number of antenatal appointments [30], i.e. a number comparable to second pregnancies with a similar risk. In reality, the reduced number of antenatal appointments for low-risk nulliparous women would be challenging to implement in certain countries, as it would require quite a significant change in the system by which midwives and clinicians are used to manage pregnancies.

For the present study we did not undertake a headroom analysis for maximum additional cost of the new test strategy to be considered cost-effective under certain willingness to pay thresholds [70, 71]. This might be an interesting option for future research as it could provide further insight for the test developers regarding the further development of the test [70]. However, in our case, due to uncertainties both in test accuracy as well as willingness to pay threshold, it was not found instructive to perform a headroom analysis at this moment. Nevertheless, in order to account for these uncertainties, the current study design employed exploratory scenario analysis, based on plausible ranges of sensitivity and specificity provided by expert opinion, and focuses on the exploration of accuracy scenarios for a new test strategy and assess at which incremental cost the new screening test could still be cost-effective, using a fixed price. Due to limited previous research exploring the cost-effectiveness of preeclampsia screening, we did not have any reference threshold as to the ICER per preeclampsia case averted that would be regarded as cost-effective. Therefore we used various willingness to pay thresholds as applied in a previous study [14], to explore the range of plausible thresholds for all four participating countries.

In conclusion, in this assessment of cost-effectiveness of early screening for preeclampsia, we have shown that there were some general important parameters that drive the cost-effectiveness in the four participating countries. Compared to the current situation, the scenario analyses showed that the new screening test can be cost saving in the UK. In The Netherlands, Ireland, and Sweden, the cost-effectiveness of the new test strategy would depend on the acceptable willingness to pay threshold per preeclampsia cases averted. Further economic evaluation studies and long-term follow-up based on proven accuracy of the test will reveal whether the new screening test for preeclampsia can truly be a cost-effective option compared to the current situation.

## Supporting information

S1 Table Survey for healthcare professionals. (PDF)

S2 Table CHEERS checklist. (PDF)
