# Supplementary material for: Early cost-effectiveness analysis of screening for preeclampsia in nulliparous women: A modelling approach in European high-income settings
Source: PLoS One. 2022 Apr 21;17(4):e0267313. doi: 10.1371/journal.pone.0267313 (PMC9022877; doi:10.1371/journal.pone.0267313)
Supplement: S1 Table — (PDF) [file pone.0267313.s001.pdf]

Early Cost-Effectiveness Analysis of Screening for Preeclampsia in Nulliparous Women: A Modelling Approach in European High-Income Settings

Neily Zakiyah [1,2,3], Robin Tuytten [4], Philip N. Baker [5], Louise C. Kenny [6], Maarten J. Postma [1,3,7,8], Antoinette D.I van Asselt [1,7,9] on behalf of IMPROvED Consortium

Running title: Early CEA of Screening for Preeclampsia in European High-Income Settings

[1] Unit of PharmacoTherapy, - Epidemiology & -Economics (PTE2), Department of Pharmacy, University of Groningen, Groningen, The Netherlands.

[2] Department of Pharmacology and Clinical Pharmacy, Faculty of Pharmacy, Universitas Padjadjaran, Bandung, Indonesia

[3] Center of Excellence in Higher Education for Pharmaceutical Care Innovation, Universitas Padjadjaran, Bandung, Indonesia

[4] Research & Development, Metabolomic Diagnostics, Little Island, Ireland

[5] College of Life Sciences, University of Leicester, United Kingdom

[6] Department of Women's and Children's Health, the Faculty of Health and Life Sciences, University of Liverpool, United Kingdom.

[7] Unit of Global Health, Department of Health Sciences, University of Groningen, University Medical Center Groningen, , Groningen, The Netherlands

[8] Department of Economics, Econometrics & Finance, Faculty of Economics & Business, University of Groningen, Groningen, The Netherlands

[9] Unit of Patient Centered Health Technology Assessment, Department of Epidemiology, , University of Groningen, University Medical Center Groningen, Groningen, The Netherlands

IMPROvED questionnaire for healthcare providers

Part I Usual care in normal pregnancy

1. Could you indicate, in percentages, in what way pregnant women (nulliparous, healthy, no risk factors) are managed in your country? Options are obstetrician (either or not in collaboration with for instance a midwife), clinical midwife, and community based midwife or GP.
please enter percentage as a whole number ranging between 0 and 100 and make sure the different percentages add up to 100 *

- [ ] percentage managed by an obstetrician (with or without other health care workers)
- [ ] percentage managed by a clinical midwife
- [ ] percentage managed by a community midwife or GP

2. What is the average pregnancy duration/gestational age (in **weeks**) at which women come in for the booking visit?

- [ ] obstetrician (with/without other healthcare workers)
- [ ] clinical midwife
- [ ] community midwife or GP

3. What is the frequency of check-ups during (normal) first pregnancies? there is room for 4 different frequencies, if you only need three, for instance, you can start with the initial fequency, then use one of the middle frequencies, and finally fill out the ultimate frequency. Please also mention the gestational age up to which the frequency applies.

	initially every .... weeks (frequency)	until .... weeks (gestational age)	then every .... weeks (frequency)	until .... weeks (gestational age)	then every .... weeks (frequency)	until .... weeks (gestational age)	ultimately every ... weeks (frequency)
obstetrician (with/without other health care workers)							
clinkcal midwife							
community midwife or GP							

4. how long does a standard visit take, in minutes?

	booking visit .... minutes	follow-up visit .... minutes
obstetrician (with/without other health care workers)		
clinical midwife		
community midwife or GP		

5. for pregnancies managed by an obstetrician (with or without other health care workers), a regular visit consists of:
if a certain test is performed at each visit, please check 'always', otherwise fill out the frequency in one of the other columns

	always	first/booking visit only	only every .... visit	only at .... weeks (multiple time points possible)	other frequency (please specifiy)
measuring blood pressure	☐	☐			

weighing	☐	☐			
fetal heart rate auscultation	☐	☐			
blood test for Hb	☐	☐			
blood test for rubella	☐	☐			
blood test for HIV	☐	☐			
blood test for treponema pallidum	☐	☐			
blood test for blood group and irregular erythrocyte	☐	☐			
blood test for other	☐	☐			
urine test for protein	☐	☐			

urine test for other	☐	☐			
ultrasound	☐	☐			
cardiotocography	☐	☐			
other	☐	☐			

6. for pregnancies managed by a clinical midwife, a regular visit consists of:

if a certain test is performed at each visit, please check 'always', otherwise fill out the frequency in one of the other columns

	always	first/booking visit only	only every .... visit	only at .... weeks (multiple time points possible)	other frequency (please specifiy)
measuring blood pressure	☐	☐			
weighing	☐	☐			
fetal heart rate auscultation	☐	☐			
blood test for Hb	☐	☐			

blood test for rubella	☐	☐			
blood test for HIV	☐	☐			
blood test for treponema pallidum	☐	☐			
blood test for blood group and irregular erythrocyte	☐	☐			
blood test for other	☐	☐			
urine test for protein	☐	☐			
urine test for other	☐	☐			
ultrasound	☐	☐			
cardiotocography	☐	☐			

other	☐	☐			

7. for pregnancies managed by a community midwife or a GP, a regular visit consists of:

if a certain test is performed at each visit, please check 'always', otherwise fill out the frequency in one of the other columns

	always	first/booking visit only	only every .... visit	only at .... weeks (multiple time points possible)	other frequency (please specifiy)
measuring blood pressure	☐	☐			
weighing	☐	☐			
fetal heart rate auscultation	☐	☐			
blood test for Hb	☐	☐			
blood test for rubella	☐	☐			
blood test for HIV	☐	☐			
blood test for treponema pallidum	☐	☐			

blood test for blood group and irregular erythrocyte	☐	☐			
blood test for other	☐	☐			
urine test for protein	☐	☐			
urine test for other	☐	☐			
ultrasound	☐	☐			
cardiotocography	☐	☐			
other	☐	☐			

Part II Usual care for pregnant women considered at high risk for pre-eclampsia

8. What is the typical/average number of weeks at which the patient is recognized to be at high risk for pre-eclampsia? *

- ..... weeks (fill out in whole weeks)
- in my country, there is no official 'high risk' status, pre-eclampsia is usually only recognized when signs are detected

9. What percentage of pregnant women considered to be at **high risk** for developing pre-eclampsia (either from the start, or later in pregnancy) are managed by the obstetrician, and will there be cases still managed by a midwife? *please enter percentage as a whole number ranging between 0 and 100 and make sure the different percentages add up to 100* *

- percentage managed by an obstetrician (with or without other health care workers)
- percentage managed by a clinical midwife
- percentage managed by a community midwife or a GP

10. What is the frequency of check-ups during these **high-risk** pregnancies? there is room for 4 different frequencies, if you only need three, for instance, you can start with the initial fequency, then use one of the middle frequencies, and finally fill out the ultimate frequency. Please also mention the gestational age up to which the frequency applies.

	initially every .... weeks (frequency)	until .... weeks (gestational age)	then every .... weeks (frequency)	until .... weeks (gestational age)	then every .... weeks (frequency)	until .... weeks (gestational age)	ultimately every ... weeks (frequency)
obstetrician (with or without other health care workers)							
clinical midwife							
community midwife or GP							

11. how long does a visit in a **high-risk** pregnancy take, in minutes? please note that if high-risk pregnancies are only treated by an obstetrician, you do not need to fill out the other two categories

	minutes
obstetrician (with or without other health care workers)	
clinical midwife	
community midwife or GP	

12. for **high-risk** pregnancies managed by an obstetrician (with or without other health care workers), a regular visit consists of:
if a certain test is performed at each visit, please check 'always', otherwise fill out the frequency in one of the other columns

	always	only every .... visit	only at .... weeks (multiple time points possible)	other frequency (please specifiy)
measuring blood pressure	☐			

measuring blood pressure	☐			
weighing	☐			
fetal heart rate auscultation	☐			
blood test for Hb	☐			
blood test for rubella	☐			
blood test for HIV	☐			
blood test for treponema pallidum	☐			
blood test for blood group and irregular erythrocyte	☐			
blood test for other	☐			
urine test for protein	☐			

urine test for other	☐			
ultrasound	☐			
cardiotocography	☐			
other	☐			

13. for **high-risk** pregnancies managed by a clinical midwife, a regular visit consists of:

if a certain test is performed at each visit, please check 'always', otherwise fill out the frequency in one of the other columns

	always	only every .... visit	only at .... weeks (multiple time points possible)	other frequency (please specifiy)
measuring blood pressure	☐			
weighing	☐			
fetal heart rate auscultation	☐			
blood test for Hb	☐			

blood test for rubella	☐			
blood test for HIV	☐			
blood test for treponema pallidum	☐			
blood test for blood group and irregular erythrocyte	☐			
blood test for other	☐			
urine test for protein	☐			
urine test for other	☐			
ultrasound	☐			
cardiotocography	☐			

other	☐			

14. for **high-risk** pregnancies managed by a community midwife or GP, a regular visit consists of:

if a certain test is performed at each visit, please check 'always', otherwise fill out the frequency in one of the other columns

	always	only every .... visit	only at .... weeks (multiple time points possible)	other frequency (please specifiy)
measuring blood pressure	☐			
weighing	☐			
fetal heart rate auscultation	☐			
blood test for Hb	☐			
blood test for rubella	☐			
blood test for HIV	☐			
blood test for treponema pallidum	☐			

blood test for blood group and irregular erythrocyte	☐			
blood test for other	☐			
urine test for protein	☐			
urine test for other	☐			
ultrasound	☐			
cardiotocography	☐			
other	☐			

15. Would you prescribe any prophylactic medication in a **high-risk** pregnancy?

- ○ no
- ○ yes

What prophylactic drugs would you prescribe?

- ☐ acetylsalicylic acid, enter mg per day
- ☐ calcium, enter mg per day
- ☐ other, please specify
- ☐ enter mg per day of other drug
- ☐ other (2), please specify
- ☐ enter mg per day of other (2) drug

16. Would you prescribe any therapeutic medication in a **high-risk** pregnancy?

- ○ no
- ○ yes

What therapeutic drugs would you prescribe?

- ☐ acetylsalicylic acid, enter mg per day
- ☐ calcium, enter mg per day
- ☐ other, please specify
- ☐ enter mg per day of other drug
- ☐ other (2), please specify
- ☐ enter mg per day of other (2) drug

17. Is there any prophylactic or therapeutic action besides intensified monitoring or medication that you would advise or prescribe to a high-risk patient?

- ○ No
- ○ Yes, please specify:

Part III Treatment of pre-eclampsia

18. After onset of pre-eclampsia, where are patients usually treated?

- women diagnosed with pre-eclampsia are always admitted to hospital until after delivery
- some of the women diagnosed with pre-eclampsia are treated as outpatients, and some are admitted

what percentage of women diagnosed with pre-eclampsia is treated as outpatient, and what percentage is treated as inpatient?

	percentage (please enter a whole number ranging between 0 and 100) *
outpatient *	
inpatient *	

19. during hospital admission, how are pre-eclampsia patients typically treated? please check those items that are part of treatment and then enter the frequency per week - or check the box when something is performed every day

	daily	.... times per week
blood pressure measurements	☐	
urine assessment	☐	
weight	☐	
fetal heart rate monitoring by cardiotocography	☐	
fetal heart rate monitoring by auscultation	☐	
full blood count	☐	
blood tests of renal function	☐	
blood tests of liver function	☐	
ultrasound assessment of fetal growth/wellbeing	☐	
$MgSO_4$ medication	☐	
antihypertensive medication	☐	
other medication	☐	
admission to a high-care unit (please check the 'always' box if this is a yes)	☐	
other	☐	

20. please specify, following the previous question:
unless you have checked one of these options (other medication, admission to high-care unit, or other) in the previous question, you do not need to answer this

if you indicated any 'other' medication, please specify	
if admission to a high-care unit, what would be the indication	
if any 'other' diagnostic/therapeutic action, what is it	

21. Could you estimate, the proportion of patients with expectant or conservative management (as opposed to those with active management or immediate delivery)? Please enter a whole number ranging between 0 and 100

22. Could you estimate, the average gestational age (in whole weeks) at which the baby is delivered in pregnancies complicated by pre-eclampsia?

23. Could you estimate, for your country, the proportion of deliveries that are preceded by induction of labour and the proportion of C-sections in **normal pregnancies** for various categories of gestational age?

	% induced delivery in normal pregnancies	% C-sections in normal pregnancies
gestational age 34-37 weeks		
gestational age 37-42 weeks		

24. Could you estimate, for your country, the proportion of deliveries that are preceded by induction of labour and the proportion of C-sections in **pre-eclampsia pregnancies** for various categories of gestational age?

	% induced delivery in pre-eclampsia pregnancies	% C-sections in pre-eclampsia pregnancies
gestational age <24 weeks		
gestational age 24-28 weeks		
gestational age 28-32 weeks		
gestational age 32-34 weeks		
gestational age 34-37 weeks		
gestational age 37-42 weeks		

25. what is the typical length of stay (in days) in hospital after a normal delivery and after a C-section, for **normal pregnancies**? please note there are separate entries for mothers and babies as length of stay can differ between these

	normal delivery - length of stay mother	normal delivery - length of stay baby	C-section - length of stay mother	C-section - length of stay baby
gestational age 34-37 weeks				
gestational age 37-42 weeks				

26. what is the typical length of stay (in days) in hospital after a normal delivery and after a C-section, for **pre-eclampsia pregnancies**? please note there are separate entries for mothers and babies as length of stay can differ between these

	normal delivery - length of stay mother	normal delivery - length of stay baby	C-section - length of stay mother	C-section - length of stay baby
gestational age <24 weeks				
gestational age 24-28 weeks				
gestational age 28-32 weeks				
gestational age 32-24 weeks				
gestational age 34-37 weeks				
gestational age 37-42 weeks				

General questions

27. what is your estimate of the prevalence of pre-eclampsia in nulliparous pregnancies in your country? (either the official number, or if that is not available, a 'best guess') please enter a whole number ranging between 0 and 100

28. what is your guess on the percentage of privately insured patients among the population in question (nulliparous pregnant women)? please enter a whole number ranging between 0 and 100

29. is there a (publicly available) guideline for the **general management of pregnancy** in your country? If so, could you fill out where to find it (if possible, a website-address)

30. is there a (publicly available) guideline for the **management of pre-eclampsia and eclampsia** in your country? If so, could you fill out where to find it (if possible, a website-address)

31. I am:

- an obstetrics nurse
- a clinical midwife
- a community midwife
- a GP
- an obstetrician in a general hospital
- an obstetrician in a teaching hospital
- an obstetrician in a university hospital
- other - please specify

*

32. I practice in:

- Germany
- Ireland
- Sweden
- The Netherlands
- UK
- Other - please specify
